# A Nonlinear Transform-Based Variability Index CFAR Detector for Doppler-Extended Targets

**DOI:** 10.3390/s26061931

**Published:** 2026-03-19

**Authors:** Lin Cao, Yuxin He, Zongmin Zhao, Chong Fu, Dongfeng Wang

**Affiliations:** 1 Center for Target Cognition Information Processing Science and Technology, Beijing Information Science and Technology University, Beijing 100101, China; charlin@bistu.edu.cn (L.C.); 2023020534@bistu.edu.cn (Y.H.); 2 School of Information and Communication Engineering, Beijing Information Science and Technology University, Beijing 100101, China; 3 School of Computer Science and Engineering, Northeastern University, Shenyang 110169, China; fuchong@mail.neu.edu.cn; 4 Beijing TransMicrowave Technology Company, Beijing 100192, China; wdf@invo.cn

**Keywords:** frequency-modulated continuous-wave (FMCW) radar, constant false alarm rate (CFAR) detector, Doppler-extended target (DET), range-Doppler matrix (RDM)

## Abstract

In frequency-modulated continuous-wave (FMCW) radar systems, the detection of Doppler-extended targets (DETs) is a critical challenge. The micro-Doppler effects induced by the motion of extended targets such as pedestrians cause the echo energy to spread along the Doppler dimension. As a result, a single range-Doppler cell is unlikely to form a pronounced amplitude peak above the background noise level. Consequently, existing constant false alarm rate (CFAR) methods that rely on single-cell amplitude decisions tend to suffer from performance degradation in DET scenarios and exhibit limited adaptability under varying clutter conditions. To solve these issues, we propose a nonlinear transform–based variability index CFAR detector for DET (DET-NTVI-CFAR), with the aim of improving detection probability and maintaining stable false alarm control in complex clutter backgrounds. This work constructs a detection statistic by applying a nonlinear transform to the accumulated power cells and derives the threshold from the corresponding probability distribution model. A variability index CFAR (VI-CFAR) decision strategy is introduced to select the appropriate detection branch under different operating conditions. In the threshold design stage, the false alarm probability expressions of three sub-detection methods are derived to guide the selection of threshold parameters. Simulation results demonstrate that the proposed method achieves stable false alarm control and improves detection probability in various environments. Field test results also confirm the applicability of the DET-NTVI-CFAR detector.

## 1. Introduction

Target detection is a fundamental task in modern signal processing frameworks. It provides the basis for subsequent processing stages, including target tracking, behavior recognition, and multisource information fusion [1,2]. In recent years, intelligent transportation, autonomous driving, surveillance, and urban public safety applications have developed rapidly [3]. As a result, radar detection techniques are increasingly required to deliver reliable and stable detection performance [4]. Among multiple radar modes, frequency-modulated continuous-wave (FMCW) radar has high distance resolution, low power consumption, and strong anti-interference features. Owing to these advantages, FMCW radar has been widely applied in urban traffic monitoring and moving target detection fields [5,6]. Against this background, extensive efforts have been devoted to improving detection performance in FMCW radar systems.

Recent studies have investigated range-Doppler detection performance for FMCW radar systems from both analytical and data-driven perspectives. Ehsanfar et al. conducted a theoretical receiver operating characteristic (ROC) analysis of FMCW, orthogonal frequency division multiplexing (OFDM), and hybrid radar systems under the IEEE 802.11bd frame structure [7]. Their analysis is based on a two-dimensional discrete Fourier transform (2D-DFT) in the delay–Doppler domain and binary hypothesis testing. The results indicate that hybrid waveforms achieve approximately 5–7 dB detection gain compared to standalone schemes. In parallel, deep learning-based estimation and multitarget detection techniques have also been explored to enhance range-Doppler processing robustness under complex environments. Delamou et al. proposed a convolutional neural network (CNN)-based method to directly estimate target range and velocity from range-Doppler maps (RDMs) for multitarget detection [8]. Their results indicate improved estimation accuracy and reduced prediction time compared with the conventional two-dimensional (2D) periodogram, the 2DResFreq method, and the VGG-19 network across various signal-to-noise ratio (SNR) conditions. Carrera et al. investigated machine learning-based radar target detection as an alternative to conventional constant false alarm rate (CFAR) and moving target indicator (MTI) or moving target detector (MTD) algorithms [9]. The results show that deep learning improves detection performance under very low signal-to-clutter ratios while keeping the false alarm probability within predefined limits. Yavuz et al. proposed a CNN that took the range-Doppler ambiguity function as input and evaluated its performance using simulated single-target range-Doppler data [10]. The results indicate that the CNN-based detector can achieve higher detection probability and substantially reduced false alarm probability compared with the classical cell-averaging CFAR (CA-CFAR) detector. These studies highlight the importance of reliable and robust detection mechanisms in practical FMCW radar systems. At the same time, learning-based detectors typically rely on task-specific training data and often require operating-point calibration to maintain stable false alarm control under varying conditions. In contrast, statistically modeled CFAR methods regulate the false alarm probability through analytically derived thresholds and require no offline training. Nevertheless, many real-world scenarios involve extended targets, which introduce additional statistical challenges beyond conventional point-target detection.

In urban environments, pedestrians represent typical moving targets whose radar echoes exhibit Doppler extension. Unlike ideal point targets, pedestrian echoes present a continuous distribution along the Doppler dimension. Due to variations in gait periodicity, walking speed, and dynamic body motion, the target energy is spread across multiple velocity bins. This Doppler-extended behavior imposes more stringent requirements on detector design [11,12]. Under multipath propagation and low SNR conditions, the target energy is easily masked by clutter. Thus, the detection probability decreases and the false alarm probability increases [13]. Consequently, the statistical properties of clutter play a critical role in radar detection performance.

To cope with the unfavorable effects of clutter, extensive research has been devoted to the design of effective CFAR processors. From the perspective of radar detection theory, the early radar systems were limited by spatial resolution. In this case, the clutter echoes can be reasonably modeled as the superposition of a large number of independent scatterers, resulting in approximately Gaussian statistics [14]. Based on this assumption, Finn et al. introduced the CA-CFAR detector in 1968 [15]. By estimating based on the mean value of the reference cells in the sliding window, the detector estimates the local background power and uses this estimate to determine the detection threshold. Due to its simplicity and low computational complexity, CA-CFAR maintain a CFAR performance in uniform clutter environments. However, the effectiveness of this method depends heavily on the assumption that no target returns are present in the reference window and that the samples follow an independent and identically distributed (IID) distribution. These assumptions are rarely met in practical scenarios. To mitigate these limitations, several improved CFAR schemes were subsequently developed. In 1973, the greatest-of CFAR (GO-CFAR) method was proposed to tackle the issue of single-sided clutter. This method independently averages the forward and backward reference windows and selects the larger estimate to form the detection threshold [16]. On the other hand, the smallest-of CFAR (SO-CFAR), presented in 1978, mitigates the impact of target contamination in the reference cells by selecting the smaller of the two estimates [17]. While GO-CFAR demonstrates superior robustness against targets situated on the edge clutter, SO-CFAR exhibits greater sensitivity to targets on the low-clutter side. However, both techniques can introduce threshold bias in homogeneous environments, which reduces detection probability and affects false alarm stability. To improve the algorithm’s robustness in complex and multiobject scenarios, Rohling introduced the ordered-statistics CFAR (OS-CFAR) detector in 1983 [18]. This method estimates the clutter level by ranking the reference samples and using the k-th ordered value. As a result, OS-CFAR significantly improves robustness against multiple interfering targets and clutter nonuniformity. However, under homogeneous conditions, its SNR efficiency is reduced because it utilizes only a portion of the reference cells for background estimation. As radar operating environments become increasingly complex, there has been growing interest in CFAR detectors that can adapt more effectively to varying background conditions. Barboy offered the censored cell-averaging CFAR (CCA-CFAR) detector to improve the reliability of threshold estimation [19]. The method iteratively removes reference samples associated with interfering targets and retains only clutter cells whose statistical characteristics are similar to those of the cell under test (CUT). Furthermore, Michael et al. developed the variability index CFAR (VI-CFAR) detector, which uses the variability index statistic and the average ratio of the leading and trailing reference windows to classify the background. VI-CFAR adaptively selects among different CFAR structures, which improves robustness in non-uniform clutter environments [20]. However, when there are multiple targets on both sides of the reference window, data contamination aggravates the masking effect and leads to performance degradation. To address this issue, Lu et al. improved VI-CFAR by combining ordered statistics with adaptive censoring [21], which enhances robustness under complex clutter and multitarget conditions.

In recent years, research on CFAR detectors in complex clutter environments has intensified, focusing on key issues such as constructing clutter models, suppressing multitarget interference, and implementing adaptive threshold mechanisms. In scenarios with multitarget occlusion, the performance of traditional CFAR detection schemes often deteriorates significantly because they depend on background power estimation. To address this challenge, Cao et al. proposed a sparse adaptive correlation maximization CFAR (SACM-CFAR) detector, which is guided by principles of compressed sensing [22,23]. By substituting background estimation with correlation-based detection, SACM-CFAR iteratively estimates parameters by eliminating previously detected targets. This approach enhances the detectability of weak targets while maintaining effective control over false alarm probability. To manage edge clutter and heterogeneous background conditions, Orlando et al. developed a novel CFAR detector based on the expectation-maximization (EM) algorithm. The EM algorithm adaptively estimates background statistical parameters, leading to better false alarm control compared to classic schemes such as CA-CFAR, GO-CFAR, and SO-CFAR [24]. In related studies, He et al. introduced a statistical interference suppression CFAR (IS-CFAR) detector that significantly mitigates the negative effects of background nonuniformity on detection performance [25]. Within the context of VI-based CFAR detectors, Zhu et al. recommended a robust variability index CFAR (BVI-CFAR) framework that incorporates Bayesian interference control techniques. The BVI-CFAR framework utilizes background segmentation and feedback mechanisms to suppress multitarget interference in complex environments [26]. To reduce the increase in false alarm probability of VI-CFAR under Weibull-distributed clutter, Wang et al. developed a robust variation index CFAR (RWVI-CFAR) detector. This detector enhances detection capability in multitarget scenarios by introducing an automatic outlier censoring maximum likelihood CFAR (AOCML-CFAR) strategy [27]. Coluccia et al. systematically explored a k-nearest neighbors (KNN) approach to improve detection performance. In this framework, either original measurements or well-known data are employed as feature vectors and input to the KNN decision rule [28]. Additionally, Liu et al. incorporated prior clutter information into the CFAR design, achieving adaptive tracking of dynamically changing background statistics [29]. To further optimize detection stability in non-uniform clutter environments, Zhao et al. constructed a cumulative target statistic to improve detection performance under complex background conditions [30].

To resolve the challenges in Doppler-extended targets (DET) detection, several methods have been developed. By using rank-based statistical tests, Yang et al. estimated the number of scattering centers of extended targets, thereby enhancing robustness [31]. Ye et al. advocated an amplitude-weighted cross-correlation detector that achieves a higher integration gain and effectively reduces computational burden [32]. Zhang et al. proposed an enhanced detection scheme, which accumulates target units along the Doppler dimension and selects the maximum response [33]. This scheme improved the performance of DET detection and enabled long-distance pedestrian detection. In millimeter-wave radar systems, Wei et al. introduced a regionalized CFAR method, which exploits diversity gain to significantly improve detection SNR and enhance target detectability in complex environments [34]. Although these methods improve DET detection from different perspectives, their design objectives and operating assumptions differ from the framework considered in this work. Rank-test–based approaches mainly focus on estimating the number of dominant scattering centers of extended targets to support subsequent decision strategies. Such methods are primarily designed for high-resolution extended targets and do not explicitly address Doppler-domain energy aggregation with analytically controlled CFAR thresholds under heterogeneous clutter. Cross-correlation–based detectors using concurrent RDMs exploit multiple range–Doppler representations and apply amplitude-weighted correlation statistics to obtain integration gain and improve robustness. However, these approaches rely on the consistency of multiple observations and typically require scenario-dependent threshold tuning, which complicates explicit false alarm regulation across varying backgrounds. Doppler-accumulation–based enhanced detectors improve DET detectability by integrating target energy across Doppler bins, often followed by peak-selection strategies to form the detection statistic. While effective in improving integration gain, the resulting statistic may be dominated by locally strongest components when multiple extended targets overlap. Area-based CFAR methods extend the CUT from a single cell to a two-dimensional region in the range–Doppler plane and exploit diversity gain to improve detection SNR in complex environments. Nevertheless, their performance depends on region size selection and assumptions on background homogeneity. These limitations motivate the development of a DET detection framework that can effectively exploit Doppler-domain energy aggregation while maintaining explicit and analytically tractable false alarm control under heterogeneous clutter conditions.

Motivated by the above, this paper proposes a new CFAR detector tailored for DET, and systematically investigates its design principles and performance characteristics. The analytical derivations in this paper are developed under the exponential clutter assumption, which corresponds to the special case of Weibull clutter with a shape parameter equal to one. The performance of the proposed detector in more complex scenarios, such as interference and edge clutter environments, is evaluated through simulations. The main contributions of this work are summarized below:

(1) A nonlinear transform-based variability index CFAR detector for DET (DET-NTVI-CFAR) is proposed. This detector is developed based on the VI-CFAR framework but specifically tailored to the DET reflection model. The proposed detector enhances the tail characteristics of the amplitude distribution by applying a nonlinear transform to accumulated power cells along the Doppler dimension. Furthermore, by incorporating the VI-CFAR strategy, the detector not only achieves a high detection probability in homogeneous environments but also exhibits strong performance in interference and edge clutter environments.
(2) This paper discusses three detection methods used in DET-NTVI-CFAR: the DET Transform Cell Average CFAR (DET-TCA-CFAR), the DET Transform Smallest-of CFAR (DET-TSO-CFAR), and the DET Transform Greatest-of CFAR (DET-TGO-CFAR). It presents the corresponding threshold factors and expressions for false alarm probability. Additionally, the threshold for the DET-NTVI-CFAR detector is calculated adaptively using the VI decision mechanism.
(3) The performance of the proposed detector is evaluated through simulations and field tests. Monte Carlo simulations were performed to investigate the influence of key parameters on detection performance, with comparative evaluation against other CFAR methods across three distinct environments. Field tests on an FMCW radar system platform further confirmed the practical effectiveness of the detector. The results show that the proposed method offers more continuous and denser target detections for DET.

## 2. DET Signal Model and FMCW Radar Signal Processing

### 2.1. DET Signal Model

In FMCW radar systems, a target may exhibit non-rigid motion, or a group of targets may possess an inherent velocity distribution. In such cases, the received echoes no longer manifest as a single Doppler frequency component. Instead, they spread over multiple Doppler bins.

Consider a typical FMCW radar transmit signal, whose equivalent complex baseband representation can be expressed as:

(1) $$s_{tx}\left(t\right)=\exp\left\{j2\pi \left(f_{0}t+\frac{B}{2T_{c}}t^{2}\right)\right\},t\in [0,T_{c})$$

where $f_{0}$ denotes the carrier frequency, B is the modulation bandwidth, and $T_{c}$ represents the duration of a single chirp.

For an extended target with a velocity distribution, the received echo can be modeled as a superposition of multiple scattering components, given by

(2) $$S_{r}\left(t\right)=\sum_{p=1}^{P}\alpha_{p}\exp\left\{j2\pi \left[f_{0}(t-\tau_{p})+\frac{B}{2T_{c}}(t-\tau_{p}{)}^{2}\right]\right\}$$

where $\alpha_{p}$ and $\tau_{p}$ denote the complex amplitude and propagation delay of the *p*-th scattering component, respectively. Within a single chirp, the target displacement is assumed to be sufficiently small such that the Doppler effect can be neglected, and the motion-induced phase variation is mainly reflected across successive chirps.

After dechirping and low-pass filtering, the baseband signal associated with the l-th chirp can be approximately written as:

(3) $$x_{l}\left(t\right)=\sum_{p=1}^{P}\alpha_{p}\exp\left\{j2\pi \left(f_{b,p}t+f_{d,p}lT_{r}\right)\right\}$$

where $f_{b,p}=\frac{B}{T_{c}}\tau_{p}$ denotes the beat frequency associated with range, $f_{d,p}=\frac{2v_{p}}{\lambda }$ is the Doppler frequency related to the radial velocity $v_{p}$ of the p-th scatterer, λ is the carrier wavelength, and $T_{r}$ denotes the chirp repetition interval. Here, t represents the fast-time variable, while l is the slow-time chirp index.

When the target exhibits a velocity distribution $v_{p}\in \left[v_{min},v_{max}\right]$, the target energy spreads over multiple Doppler bins after performing a discrete Fourier transform along the slow-time dimension. The corresponding Doppler power spectrum can be expressed as:

(4) $$S\left(f_{d}\right)=\sum_{p=1}^{P}|\alpha_{p}{|}^{2}\delta \left(f_{d}-\frac{2v_{p}}{\lambda }\right)$$

where $f_{d}$ denotes the Doppler frequency variable. In practical FMCW radar systems, the Doppler power spectrum of an extended target appears as a continuous spectral envelope due to finite observation windows and spectral leakage. To quantitatively characterize the degree of Doppler extension, the Doppler spread length of the target is defined as:

(5) $$L_{DET}=\left\lfloor \frac{f_{d,max}-f_{d,min}}{\Delta f_{d}}\right\rfloor$$

where $f_{d,max}$ and $f_{d,min}$ denote the maximum and minimum Doppler frequencies occupied by the target, respectively. $\Delta f_{d}=\frac{1}{{LT}_{r}}$ represents the Doppler frequency resolution determined by the number of accumulated chirps L and the pulse repetition interval $T_{r}$, and $\left\lfloor \cdot \right\rfloor$ denotes the floor operator. Here, L denotes the number of accumulated chirps used for Doppler processing, whereas N is exclusively used to denote the number of CFAR reference cells.

In the range-Doppler domain, the received power statistic at the *u*-th range cell and the *v*-th Doppler cell can be expressed as:

(6) $$Z\left(u,v\right)=\sum_{i=1}^{L_{DET}}|X\left(u,v+i\right){|}^{2}$$

which indicates that the detection of DET inherently requires joint modeling and statistical processing of multiple correlated Doppler cells.

Existing studies have shown that directly applying conventional single-cell CFAR detectors under Doppler extension conditions violates the IID assumption of reference cells, resulting in false alarm probability mismatch. Based on the Doppler-extended signal model, incorporating accumulation strategies over extended Doppler cells together with adaptive threshold design is essential for achieving stable constant false alarm probability detection.

### 2.2. FMCW Radar Signal Processing

Target detection in FMCW millimeter-wave radar systems typically consists of three main stages: signal acquisition, chirp-level preprocessing, and CFAR detection. Firstly, the radar transmit antenna continuously emits linear frequency-modulated (LFM) waveforms. When these signals are reflected by target surfaces, the echoes are received by the radar receive antenna. The received signals are mixed with local oscillator (LO) signals in a mixer to generate beat frequencies. Following low-pass filtering to eliminate high-frequency components, we can obtain an intermediate-frequency (IF) signal containing both range and velocity information. The IF signal is sampled by the analog-to-digital converter (ADC) to produce a digital signal for subsequent processing.

During the chirp processing stage, we apply static clutter suppression to each received chirp signal to mitigate strong zero-Doppler echoes from stationary scatterers. Common approaches to suppress static clutter include zero-velocity channel nulling, moving target indication (MTI), and phase averaging cancellation (PAC). After clutter mitigation and other preprocessing steps, a one-dimensional Fast Fourier Transform (FFT) is applied along the fast-time axis to obtain the range spectrum. Following this, a Doppler FFT is performed along the slow-time dimension, resulting in a two-dimensional RDM. Each cell in the RDM represents the signal power corresponding to a specific range bin and Doppler frequency. The generated RDM is then fed into the detection stage for target decision.

## 3. Proposed Algorithm

**Figure 1:**
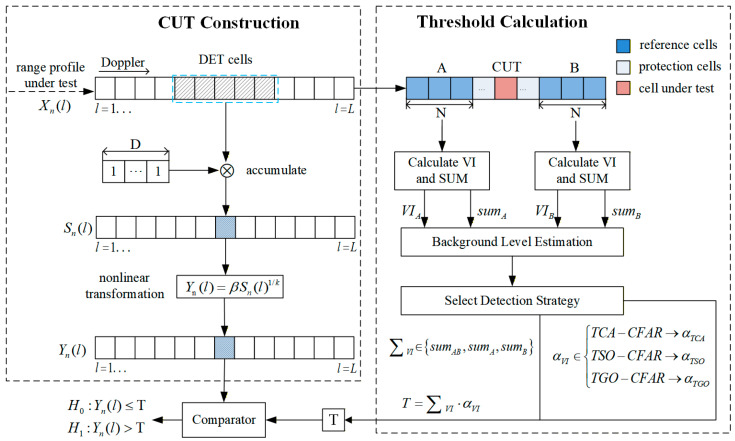
Proposed DET-NTVI-CFAR detector.

To enhance pedestrian detection performance in FMCW radar systems, this section proposes a high-accuracy and robust DET-NTVI-CFAR detector. The detector applies a nonlinear transformation to the accumulated power cells to enhance the tail characteristics of the distribution. This transformation improves the distinguishability between the target and noise and increases the detection probability. Figure 1 illustrates the structure of the proposed DET-NTVI-CFAR detector.

Within the CFAR detection framework, the overall processing procedure of the proposed DET-NTVI-CFAR consists of two main stages. In the CUT construction stage, a nonlinear transformation is applied to the accumulated power cells, and the resulting statistic is adopted as the CUT. During the threshold calculation phase, a sliding window is constructed around the CUT. The central region of the window is designated as a protection cell to prevent target energy leakage from affecting the background estimation, while the cells on both sides of the CUT are used as reference cells for background power estimation. For the forward and backward reference windows, the VI and the corresponding accumulated power statistics are calculated for background level estimation. The theoretical false alarm probability expressions associated with the three sub-detection methods involved in DET-NTVI-CFAR are derived. Subsequently, a VI-based decision mechanism is employed to adaptively select the noise power estimate and the threshold factor, and the final detection threshold is obtained through a multiplicative operation. Finally, the presence of a DET is determined by comparing the CUT statistic with the calculated threshold. The specific description is as follows.

### 3.1. CUT Construction

In DET scenarios, the target echo energy exhibits a continuous distribution along the Doppler dimension rather than being concentrated in a single range-Doppler cell. If a single-cell power statistic is directly used as the CUT, it is often difficult to produce a pronounced peak above the background noise level, especially under low SNR conditions. As a consequence, detection performance deteriorates when applying point-target CFAR schemes to DET scenarios. Therefore, it is necessary to introduce a Doppler-domain energy integration mechanism to construct a detection statistic that better captures the DET returns.

Motivated by this consideration, a sliding summation with length D is performed along the Doppler dimension of $X_{n}\left(l\right)$. This operation corresponds to integrating the energy of D adjacent Doppler cells, resulting in the statistic shown below:

(7) $$S_{n}\left(l\right)=\sum_{i=0}^{D-1}X_{n}\left(l-i\right)$$

The accumulation operation effectively integrates dispersed energy when the target is present, enhancing the effective SNR of statistical measurements. Under the noise hypothesis, since each original power cell follows an exponential distribution, the accumulated statistic $S_{n}\left(l\right)$ follows a Gamma distribution, which establishes a tractable statistical foundation for subsequent threshold design.

After Doppler-domain energy integration, a monotone nonlinear power transform is introduced to further enhance the statistical separability between target and clutter in the tail region of the distribution. The final CUT statistic is defined as

(8) $$Y_{n}(l)=\beta S_{n}(l{)}^{\frac{1}{k}}$$

where k denotes the nonlinear exponent and β is a normalization coefficient. This operation is intended to enhance the separability between the target and the background in the tail region of the distribution, because CFAR decisions are primarily governed by tail probability characteristics. In heterogeneous clutter or interference environments, Doppler-domain energy integration alone may not produce sufficiently pronounced tail contrast, thereby weakening the discrimination of weak targets. To address this issue, a nonlinear transform is introduced to expand the dynamic range of high-amplitude samples, thus amplifying tail differences and improving detectability. In particular, when 0<k<1, the transform applies a stronger amplification to larger-amplitude samples, making the target return more prominent relative to the background noise in the tail region. Since the threshold can still be constrained by tail probabilities, this nonlinear processing improves the detection probability of weak DETs while maintaining a controllable false alarm probability. From a statistical perspective, the accumulated statistic $S_{n}\left(l\right)$ follows a Gamma distribution. The transform defined in (8) is a monotone mapping. Therefore, the probability density function (PDF) and cumulative distribution function (CDF) of $Y_{n}(l)$ can be derived through a standard variable transformation. This enables an explicit relationship between the false alarm probability and the threshold factor, ensuring analytically tractable CFAR threshold design. If the order is reversed, the resulting statistic may no longer admit a closed-form distribution under the noise-only condition and make the analytical design of the CFAR threshold more complex.

In addition, the normalization coefficient β serves as a scale factor for the transformed CUT statistic. Since the accumulated statistic $S_{n}\left(l\right)$ follows a Gamma distribution under noise-only conditions, the nonlinear transformation modifies its scale characteristics. The coefficient β is introduced to ensure dimensional consistency and to provide flexible amplitude normalization of the transformed statistic, without altering the distribution family or its shape parameters. It is important to note that β does not affect the CFAR property of the detector. For a given design false alarm probability, the threshold factor can be re-parameterized to absorb the effect of β, resulting in the same false alarm probability under noise-only conditions. Consequently, β changes only the numerical scale of the detection threshold but does not influence the false alarm control mechanism. In this work, for analytical clarity and without loss of generality, β is set to unity in the simulations.

The DET detection problem can be described as a binary hypothesis test. $H_{0}$ corresponds to the hypothesis that the CUT holds only noise, and $H_{1}$ corresponds to the hypothesis that the CUT contains a target, i.e.,

(9) $$\left\{\begin{array}{l} H_{0}:x(t)=n(t),\;\;\;\;\;\;\;\;\;\;\;\;\;\;\;\;\;\;\;\;\;if\;\;\;Y_{n}(l)\leq T\\ H_{1}:x(t)=n(t)+s(t),\;\;\;\;\;\;\;\;if\;\;\;Y_{n}(l)>T \end{array}\right.$$

where T denotes the decision threshold. For comparison and decision, the PDF of $Y_{n}\left(l\right)$ needs to be obtained first. From the result in (8), we know that $Y_{n}\left(l\right)$ is derived from $S_{n}\left(l\right)$ through a nonlinear power transformation. Therefore, the PDFs of $Y_{n}\left(l\right)$ under $H_{0}$ and $H_{1}$ are given, respectively.

(10) $$f_{Y_{n}(l)\left|H_{0}\right.}\left(y\right)=\frac{k}{\beta \lambda^{D}\Gamma \left(D\right)}{\left(\frac{y}{\beta }\right)}^{kD-1}\exp\left[-\frac{1}{\lambda }{\left(\frac{y}{\beta }\right)}^{k}\right],y>0$$

(11) $$f_{Y_{n}(l)\left|H_{1}\right.}\left(y\right)=\frac{k}{\beta {\left[\lambda \left(1+SNR\right)\right]}^{D}\Gamma \left(D\right)}{\left(\frac{y}{\beta }\right)}^{kD-1}\exp\left[-\frac{1}{\lambda \left(1+SNR\right)}{\left(\frac{y}{\beta }\right)}^{k}\right],y>0$$

where *SNR* is signal-to-noise ratio. Since D is a positive integer, the CDF of $Y_{n}(l)$ can be written as:

(12) $$F_{Y_{n}(l)\left|H_{0}\right.}\left(y\right)=F_{S}\left((\frac{y}{\beta }{)}^{k}|H_{0}\right)=\frac{1}{\Gamma \left(D\right)}\gamma \left(D,\frac{1}{\lambda }{\left(\frac{y}{\beta }\right)}^{k}\right)$$

(13) $$F_{Y_{n}(l)\left|H_{1}\right.}\left(y\right)=F_{S}\left((\frac{y}{\beta }{)}^{k}|H_{1}\right)=\frac{1}{\Gamma \left(D\right)}\gamma \left(D,\frac{1}{\lambda \left(1+SNR\right)}{\left(\frac{y}{\beta }\right)}^{k}\right)$$

where $\Gamma \left(D\right)$ signifies the Gamma function evaluated at *D*.

(14) $$\Gamma \left(D\right)=\int_{0}^{+\infty }t^{D-1}e^{-t}dt,D>0$$

$\gamma \left(a,b\right)$ can be evaluated as:

(15) $$\gamma \left(a,b\right)=\Gamma \left(a\right)-\Gamma \left(a,b\right)$$

(16) $$\Gamma \left(a,b\right)=\int_{b}^{+\infty }t^{a-1}e^{-t}dt=\left(a-1\right)!e^{-b}\sum_{j=0}^{a-1}\frac{b^{k}}{j!}$$

Therefore, the expression of the false alarm probability $P_{fa}$ is given by:

(17) $$\begin{array}{ll} P_{fa} & =\int_{T}^{\infty }f_{Y_{n}(l)\left|H_{0}\right.}(y)dy\\ & =1-F_{Y_{n}(l)\left|H_{0}\right.}(T)\\ & =1-\frac{1}{\Gamma (D)}\gamma \left(D,\frac{1}{\lambda }(\frac{T}{\beta }{)}^{k}\right)\\ & =\sum_{i=0}^{D-1}\frac{1}{i!}{\left(\frac{1}{\lambda }(\frac{T}{\beta }{)}^{k}\right)}^{i}\exp[-\frac{1}{\lambda }(\frac{T}{\beta }{)}^{k}] \end{array}$$

It is evident that the false alarm probability is governed by the decision threshold *T*, the distribution parameters λ, and the power exponent k. Given a prescribed false alarm probability, the threshold factor is derived from the expression of the false alarm probability, which in turn yields the decision threshold *T*. The detection probability is then computed as:

(18) $$\begin{array}{ll} P_{d} & =\int_{T}^{+\infty }f_{Y_{n}(l)\left|H_{1}\right.}(y)dy\\ & =1-F_{Y_{n}(l)\left|H_{1}\right.}(T)\\ & =1-\frac{1}{\Gamma (D)}\gamma \left(D,\frac{1}{\lambda \left(1+SNR\right)}(\frac{T}{\beta }{)}^{k}\right)\\ & =\sum_{i=0}^{D-1}\frac{1}{i!}{\left(\frac{1}{\lambda \left(1+SNR\right)}(\frac{T}{\beta }{)}^{k}\right)}^{i}\exp\left[-\frac{1}{\lambda \left(1+SNR\right)}(\frac{T}{\beta }{)}^{k}\right] \end{array}$$

The derivations in (10)–(18) are established under the standard homogeneous-background assumption commonly adopted in radar detection theory. Specifically, within the reference window, the clutter samples are modeled as statistically IID random variables with identical distribution parameters. Under noise-only conditions in a homogeneous environment, the in-phase and quadrature components of the received signal are modeled as zero-mean Gaussian random variables, leading to exponentially distributed power samples. Consequently, the sum of D independent exponential random variables follows a Gamma distribution, which justifies the accumulated-statistic formulation in (10). Since the nonlinear transformation is strictly monotonic for k>0, the probability density function of the transformed statistic can be derived using the standard change-of-variable technique, resulting in the expressions in (10)–(18). Therefore, these expressions are mathematically valid under the homogeneous clutter assumption.

It should be emphasized that the exponential model corresponds to the special case of a Weibull distribution with shape parameter equal to one. For a general Weibull distribution with shape parameter different from one, the accumulated statistic does not follow a Gamma distribution in closed form. In such cases, the analytical expressions are no longer strictly exact. However, the threshold factor can still be determined numerically based on the actual clutter statistics.

### 3.2. Threshold Calculation

To establish the adaptive detection threshold of the proposed method, it is necessary to estimate the noise level that reflects the local clutter characteristics and to derive the expression relating the false alarm probability to the threshold factor. Specifically, a sliding window is designed in this work to construct two reference cell matrices A and B by aggregating multiple power cells. The reference cell matrices $A=\left\{x_{A}\left(i\right),l-\left\lfloor D/2\right\rfloor -N<i<l-\left\lfloor D/2\right\rfloor \right\}$ and $B=\left\{x_{B}\left(i\right),l+\left\lfloor D/2\right\rfloor <i<l+\left\lfloor D/2\right\rfloor +N\right\}$ are located on both sides of the CUT, respectively. Both matrices contain N reference cells. Between them, there are $N_{P}$ protection cells spaced apart. Although a sliding accumulation is performed along the Doppler dimension in the CUT construction stage, the reference matrices *A* and *B* are formed from two disjoint Doppler index intervals separated by protection cells. Specifically, the accumulated statistics within *A* and *B* do not share overlapping Doppler samples. Under hypothesis $H_{0}$, the clutter samples are modeled as statistically IID. Therefore, since reference matrices *A* and *B* are constructed from non-overlapping Doppler cells, their accumulated statistics remain statistically independent. The accumulated powers of the cells in matrices *A* and *B* are given by:

(19) $$\left\{\begin{array}{l} sum_{A}=\sum_{i=0}^{N}x_{A}\left(i\right)\\ sum_{B}=\sum_{i=0}^{N}x_{B}\left(i\right) \end{array}\right.$$

Under the hypothesis $H_{0}$, the PDFs of $sum_{A}$ and $sum_{B}$ are given by:

(20) $$f_{sum_{A}\left|H_{0}\right.}(x)=f_{sum_{B}\left|H_{0}\right.}(x)=\frac{1}{\lambda^{N}\Gamma (N)}x^{N-1}e^{-\frac{x}{\lambda }},x>0$$

The corresponding CDFs are given by:

(21) $$F_{sum_{A}\left|H_{0}\right.}(x)=F_{sum_{B}\left|H_{0}\right.}(x)=\frac{\gamma (N,\frac{x}{\lambda })}{\Gamma (N)}$$

Based on the PDFs and CDFs of the accumulated power cells, we develop the corresponding DET-TCA, DET-TSO, and DET-TGO CFAR methods, which are used for background level estimation and decision-making. These are discussed separately in the following.

#### 3.2.1. DET-TCA-CFAR Method

The detection threshold for DET-TCA is defined as:

(22) $$T_{TCA}=\alpha_{TCA}\cdot {sum}_{AB}$$

where ${sum}_{AB}={sum}_{A}+{sum}_{B}$ denotes the estimated background noise level for the *TCA* method, and $\alpha_{TCA}$ represents the threshold factor. Once this constant $\alpha_{TCA}$ is obtained, the detection threshold can be directly computed from the above expression. To determine $\alpha_{TCA}$, the PDF under the hypothesis $H_{0}$ is first derived as:

(23) $$f_{{sum}_{AB}\left|H_{0}\right.}(x)=\frac{1}{\lambda \Gamma (2N)}{\left(\frac{x}{\lambda }\right)}^{2N-1}e^{-\frac{x}{\lambda }}$$

By combining (17), (22), and (23), the conditional false alarm probability of DET-TCA, given the reference window statistic, can be expressed as:

(24) $$\begin{array}{ll} P_{fa,TCA} & =\sum_{i=0}^{D-1}\frac{1}{i!}{\left(\frac{1}{\lambda }(\frac{T}{\beta }{)}^{k}\right)}^{i}\exp\left[-\frac{1}{\lambda }(\frac{T}{\beta }{)}^{k}\right]\\ & =\sum_{i=0}^{D-1}\frac{1}{i!}{\left(\frac{1}{\lambda }{\left(\frac{\alpha_{TCA}\cdot sum_{AB}}{\beta }\right)}^{k}\right)}^{i}\exp\left[-\frac{1}{\lambda }{\left(\frac{\alpha_{TCA}\cdot sum_{AB}}{\beta }\right)}^{k}\right] \end{array}$$

#### 3.2.2. DET-TSO-CFAR Method

The detection threshold for DET-TSO is defined as:

(25) $$T_{TSO}=\alpha_{TSO}\cdot \min({sum}_{A},{sum}_{B})$$

The PDF of $\min({sum}_{A},{sum}_{B})$ under the hypothesis $H_{0}$ is derived as:

(26) $$f_{\min({sum}_{A},{sum}_{B})\left|H_{0}\right.}(x)=\left(1-F_{{sum}_{A}\left|H_{0}\right.}(x)\right)f_{{sum}_{B}\left|H_{0}\right.}(x)+\left(1-F_{{sum}_{B}\left|H_{0}\right.}(x)\right)f_{{sum}_{A}\left|H_{0}\right.}(x)\;\;\;\;\;$$

By combining (15), (16), and (26), we obtain:

(27) $$f_{\min(sum_{A},sum_{B})\left|H_{0}\right.}(x)=\frac{2\Gamma (N,\lambda x)}{\lambda^{2}(N)}(\frac{x}{\lambda }{)}^{N-1}e^{-\frac{x}{\lambda }}$$

The conditional false alarm probability of DET-TSO, given the reference window statistic $\min(sum_{A},sum_{B})$, can be calculated as:

(28) $$\begin{array}{ll} P_{fa,TSO} & =\sum_{i=0}^{D-1}\frac{1}{i!}{\left(\frac{1}{\lambda }(\frac{T}{\beta }{)}^{k}\right)}^{i}\exp\left[-\frac{1}{\lambda }(\frac{T}{\beta }{)}^{k}\right]\\ & =\sum_{i=0}^{D-1}\frac{1}{i!}{\left(\frac{1}{\lambda }(\frac{\alpha_{TSO}\cdot \min(sum_{A},sum_{B})}{\beta }{)}^{k}\right)}^{i}\exp\left[-\frac{1}{\lambda }(\frac{\alpha_{TSO}\cdot \min(sum_{A},sum_{B})}{\beta }{)}^{k}\right] \end{array}$$

#### 3.2.3. DET-TGO-CFAR Method

The detection threshold for DET-TGO is defined as:

(29) $$T_{TGO}=\alpha_{TGO}\cdot \max({sum}_{A},{sum}_{B})$$

The PDF of $\max({sum}_{A},{sum}_{B})$ under the hypothesis $H_{0}$ is derived as:

(30) $$f_{\max({sum}_{A},{sum}_{B})\left|H_{0}\right.}(x)=F_{{sum}_{A}\left|H_{0}\right.}(x)\cdot f_{{sum}_{B}\left|H_{0}\right.}(x)+F_{{sum}_{B}\left|H_{0}\right.}(x)\cdot f_{{sum}_{A}\left|H_{0}\right.}(x)$$

By combining (15), (16), and (30), $f_{\max({sum}_{A},{sum}_{B})\left|H_{0}\right.}(x)$ can be computed as:

(31) $$f_{\max({sum}_{A},{sum}_{B})\left|H_{0}\right.}(x)=\frac{2}{\lambda \Gamma^{2}(N)}{\left(\frac{x}{\lambda }\right)}^{N-1}e^{-\frac{x}{\lambda }}\gamma (N,\frac{x}{\lambda })$$

The conditional false alarm probability of DET-TGO, given the reference window statistic $\max({sum}_{A},{sum}_{B})$, can be calculated as:

(32) $$\begin{array}{ll} P_{fa,TGO} & =\sum_{i=0}^{D-1}\frac{1}{i!}{\left(\frac{1}{\lambda }(\frac{T}{\beta }{)}^{k}\right)}^{i}\exp\left[-\frac{1}{\lambda }(\frac{T}{\beta }{)}^{k}\right]\\ & =\sum_{i=0}^{D-1}\frac{1}{i!}{\left(\frac{1}{\lambda }(\frac{\alpha_{TGO}\cdot \max(sum_{A},sum_{B})}{\beta }{)}^{k}\right)}^{i}\exp\left[-\frac{1}{\lambda }(\frac{\alpha_{TGO}\cdot \max(sum_{A},sum_{B})}{\beta }{)}^{k}\right] \end{array}$$

The above expressions describe the conditional false alarm probabilities of the DET-TCA, DET-TSO, and DET-TGO methods under their respective reference window statistics. Since the reference window statistic is a random variable, the average false alarm probability is obtained by taking the expectation of the above conditional false alarm probability over the distribution of the reference statistic. In this work, this expectation is evaluated using numerical integration.

### 3.3. Variability Index Threshold Selection Strategy

After obtaining the expression for the average false alarm probability, the detection threshold of the DET-NTVI-CFAR detector can be determined using different sub-detection strategies. In this article, the VI-CFAR strategy [20] is adopted for threshold determination, in which two parameters, namely the variability index (VI) and the mean ratio (MR), are defined and computed as follows:

(33) $$VI=1+\frac{{\hat{\sigma }}^{2}}{{\hat{\mu }}^{2}}=1+\frac{1}{N-1}\sum_{i=1}^{N}\frac{(x_{i}-x_{\mu }{)}^{2}}{(x_{\mu }{)}^{2}}$$

(34) $$MR=\frac{x_{\mu A}}{x_{\mu B}}=\frac{\sum_{i\in A}x_{i}}{\sum_{i\in B}x_{i}}$$

where ${\hat{\sigma }}^{2}$ and ${\hat{\mu }}^{2}$ denote the estimated variance and mean, respectively. The power cells within the reference window are denoted by $x_{i}$, with $x_{\mu }$ representing their arithmetic mean, while $x_{\mu A}$ and $x_{\mu B}$ denote the average values of the forward and backward sliding windows, respectively.

The selection strategy compares the VI with a preset threshold $K_{VI}$ to identify homogeneous clutter conditions and compares the MR with its threshold $K_{MR}$ to determine whether the forward and backward window means are equal [35,36], as follows:

(35) $$\left\{\begin{array}{l} VI\leq K_{VI},\;\;\;\;homogeneous\;clutter\\ VI>K_{VI},\;\;\;\;non-homogeneous\;clutter \end{array}\right.$$

(36) $$\left\{\begin{array}{l} K_{MR}^{-1}\leq MR\leq K_{MR},\;\;\;\;\;\;\;\;\;\;\;\;\;\;equal\;means\\ MR<K_{MR}^{-1}\;or\;MR>K_{MR},\;\;unequal\;means \end{array}\right.$$

Based on the comparison results of these two parameterized equations, the corresponding equivalent sub-algorithm is selected in the detection system. The DET-NTVI-CFAR scheme combines the strengths of DET-TCA-CFAR, DET-TSO-CFAR, and DET-TGO-CFAR, and computes the adaptive threshold according to the outcomes of the VI and MR tests. As a result, the DET-NTVI-CFAR detector exhibits robust performance across various environments. The decision rules for adaptive threshold calculation are summarized in Table 1.

The overall procedure of DET-NTVI-CFAR can be interpreted as a unified adaptive detection framework. After constructing the CUT, the detector evaluates the background using the VI and MR tests, and then adaptively selects an appropriate sub-algorithm (DET-TCA, DET-TSO, or DET-TGO) for threshold computation according to the rules summarized in Table 1. This unified architecture enables the detector to adjust its thresholding strategy to local clutter characteristics while preserving a statistically derived CFAR formulation.

Conventional point-target CFAR processors typically rely on single-cell CUT statistics, and their performance can degrade in DET scenarios where target energy is distributed over multiple Doppler bins. In contrast, the proposed DET-NTVI-CFAR reconstructs the CUT at the statistical-structure level. By combining Doppler-domain energy integration with tail enhancement, the constructed statistic both aggregates dispersed Doppler energy and improves target–background separability in the distribution tail. The enhanced detector adopts an accumulate-then-max strategy after Doppler accumulation, which may be more susceptible to local energy dominance when extended components from multiple targets partially overlap [33]. DET-NTVI-CFAR instead applies a nonlinear transform to the accumulated statistic and employs VI-driven branch selection, thereby mitigating threshold bias caused by interference-contaminated reference cells. The area-based CFAR (ABC) method improves the detection SNR by exploiting potential diversity gain through regional aggregation [34]. However, it does not incorporate an adaptive switching mechanism to handle interference and edge clutter conditions. As a result, its thresholding behavior may be more sensitive to local background variations, whereas DET-NTVI-CFAR can adaptively select the thresholding strategy according to Table 1, leading to more robust performance in complex backgrounds. The effectiveness of these methods is evaluated in the following section.

## 4. Simulation Analysis

In this section, the performance of the proposed DET-NTVI-CFAR detector is evaluated through numerical simulations. Unless otherwise specified, the false alarm probability is fixed at $P_{fa}=10^{-6}$ throughout the simulations. The clutter background is modeled by a Weibull distribution with a shape parameter of 1 and a scale parameter of λ=0.01. The target returns are generated according to the Swerling I model. All simulation results are obtained based on $10^{6}$ Monte Carlo trials.

In addition to reporting numerical detection performance, the simulation study is systematically organized to validate both the theoretical consistency and the structural advantages of the proposed DET-NTVI-CFAR framework. Section 4.1 first verifies the correctness of the derived false alarm probability expressions under hypothesis $H_{0}$ through Monte Carlo simulations, thereby confirming the analytical tractability of the statistical modeling. Section 4.2 then examines the parameter selection principles for the VI- and MR-based adaptive decision mechanism. Section 4.3 investigates the influence of the nonlinear exponent k, accumulation length D, and reference window size N. These parameters jointly determine the strength of tail enhancement, Doppler-domain energy integration, and the statistical stability of background estimation. Section 4.4 evaluates detection performance under homogeneous, interference, and edge clutter environments. The results demonstrate how the DET-aware CUT construction and adaptive threshold adjustment improve detection robustness compared with conventional CFAR-based approaches. Finally, Section 4.5 discusses recent artificial intelligence (AI)-based range-Doppler detection methods and clarifies the requirements for a rigorous and fair comparison with analytically designed CFAR detectors, thereby positioning the proposed framework relative to AI-based approaches.

### 4.1. Verification of Theoretical False Alarm Probability

To further validate the theoretical rigor of the derived false alarm probability expression, Monte Carlo simulations were conducted under the hypothesis $H_{0}$ for multiple parameter configurations. To facilitate fair comparison across different parameter settings, a normalized threshold scanning variable η is introduced in this subsection. The detection threshold is defined as follows:

(37) $$T=\eta T_{0}$$

(38) $$T_{0}={\beta (D\lambda )}^{\frac{1}{k}}$$

where $T_{0}$ represents the typical scale of the transformed statistic under $H_{0}$.

By combining (17), (37), and (38), the expression of the false alarm probability $P_{fa}$ is given by:

(39) $$P_{fa}=\sum_{i=0}^{D-1}\frac{1}{i!}{\left(\eta^{k}D\right)}^{i}exp(-\eta^{k}D)$$

Therefore, the false alarm probability expression can be equivalently expressed as a function of the normalized threshold scanning variable η, the nonlinear exponent k, and the accumulation window length D. This normalization eliminates the influence of scale parameters and enables fair comparison across different configurations.

**Figure 2:**
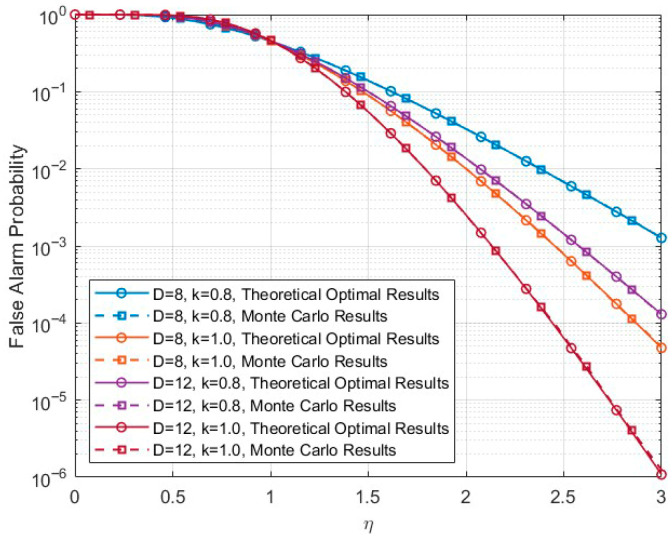
Comparison between analytical and Monte Carlo false alarm probabilities under different accumulation lengths D and nonlinear exponents k, with respect to the normalized threshold scanning variable η.

Figure 2 presents the comparison between the analytical and simulated false alarm probabilities for different accumulation lengths D=8,12 and nonlinear exponents k=0.8, 1. It can be observed that the Monte Carlo results closely coincide with the analytical predictions over the entire threshold range. Even in the low false alarm probability region, the agreement remains highly consistent. These results confirm the correctness of the derived expression and the theoretical consistency of the proposed statistical modeling framework under varying parameter conditions. Moreover, as the accumulation length D increases, the false alarm probability decays more rapidly, which is consistent with the theoretical expectation. The nonlinear exponent k also influences the distribution shape of the transformed statistic, thereby affecting the decay behavior of the false alarm probability. These observations are in full agreement with the theoretical analysis.

**Figure 3:**
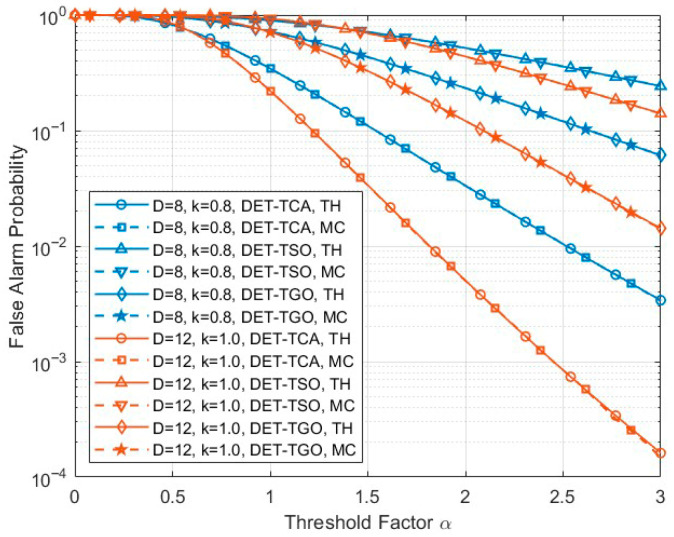
Verification of analytical and Monte Carlo false alarm probabilities for DET-TCA, DET-TSO, and DET-TGO under $H_{0}$ with respect to the threshold factor α.

Figure 3 compares the analytical and Monte Carlo false alarm probabilities of the three sub-detectors, namely DET-TCA, DET-TSO, and DET-TGO, under the hypothesis $H_{0}$. The theoretical and simulation results are denoted by “TH” and “MC”, respectively. The horizontal axis denotes the threshold factor α of each sub-detector (i.e., $\alpha_{TCA}$, $\alpha_{TSO}$, $\alpha_{TGO}$), as defined in Section 3.2. It can be observed that the Monte Carlo results closely coincide with the analytical predictions over the entire threshold range for all three methods. This consistency verifies the correctness of the derived conditional false alarm probability expressions. Moreover, as the threshold factor α increases, the false alarm probability monotonically decreases, which is consistent with the theoretical expectation of CFAR detection mechanisms. The decay behavior also varies with the accumulation length D and the nonlinear exponent k, demonstrating the influence of system parameters on the statistical distribution of the transformed detection statistic.

### 4.2. Selection of Parameters $K_{VI}$ and $K_{MR}$

The threshold settings of $K_{VI}$ and $K_{MR}$ directly affect the submethod selection mechanism of the DET-NTVI-CFAR detector under different environmental conditions, as well as its overall detection performance. In interference-free environments, $K_{VI}$ and $K_{MR}$ should be selected to ensure that the performance of DET-NTVI-CFAR closely matches that of its submethod, DET-TCA-CFAR, thereby reducing the hypothesis testing error probability. The definitions of the hypothesis testing error probabilities associated with the VI and MR decisions can be defined as:

(40) $$\theta =P\left[VI>K_{VI}|No\;Interference\right]$$

(41) $$\rho =P\left[MR<K_{MR}^{-1}\mathrm{or}MR>K_{MR}|No\;Interference\right]$$

where θ and ρ denote the probabilities of triggering the variable judgment mechanism in different decision branches of the DET-NTVI-CFAR detector.

**Figure 4:**
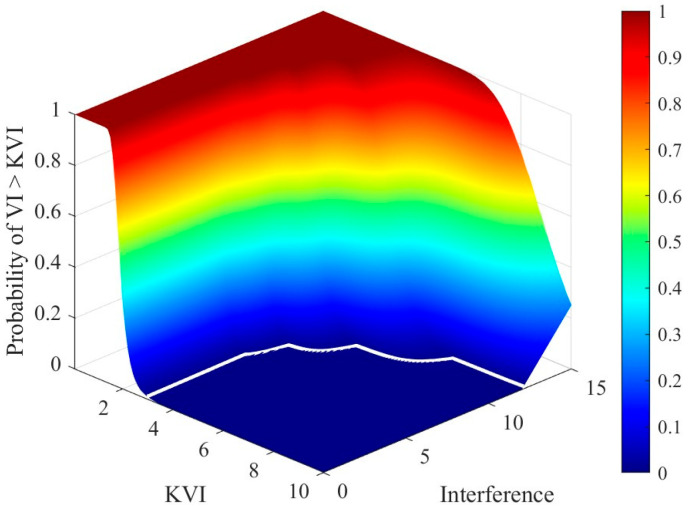
Variable-hypothesis decision probability as a function of $K_{VI}$ and interference level. The white line represents the 1% decision-probability contour.

Since the PDFs of the VI and MR are complex and not readily obtainable, Monte Carlo simulations are employed to statistically evaluate the variable decision probabilities and to guide the selection of the thresholds $K_{VI}$ and $K_{MR}$. Figure 4 presents the three-dimensional statistical results showing the probability of VI exceeding $K_{VI}$ as a function of $K_{VI}$ under different interference levels. It can be observed that the probability surface exhibits a pronounced high-probability plateau in the low-$K_{VI}$ region. As the interference intensity increases, this plateau expands toward higher values, indicating that the VI-based decision is sensitive to interference. In an interference-free environment, the probability surface remains at a low level along the interference axis. When $K_{VI}=3$, the probability of VI exceeding the threshold $K_{VI}$ is effectively constrained below 1%, satisfying the design requirement for a low false variable-decision rate. As $K_{VI}$ increases, the overall probability of triggering the variable-decision mechanism decreases. However, excessively large $K_{VI}$ values reduce the detector’s responsiveness to moderate interference variations. Conversely, smaller $K_{VI}$ values enhance sensitivity to interference but are more susceptible to false triggering caused by random noise fluctuations in homogeneous clutter. Therefore, the selection of $K_{VI}$ should strike a reasonable balance between suppressing false variable-decision triggering and maintaining effective responsiveness to interference variations.

**Figure 5:**
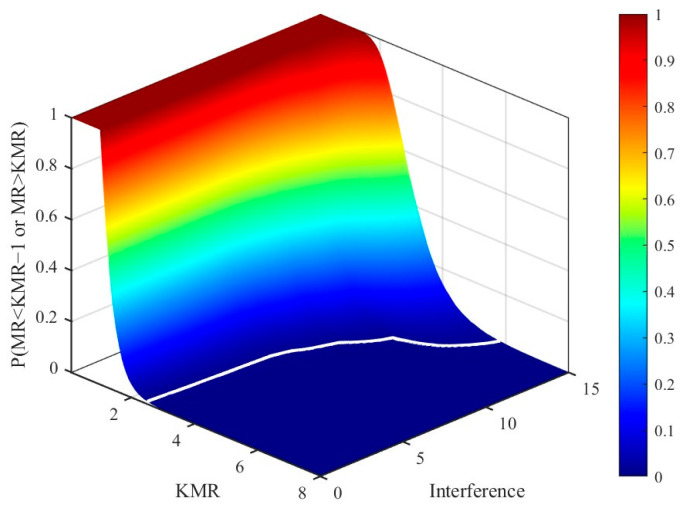
Variable-hypothesis decision probability as a function of $K_{MR}$ and interference level. The white line represents the 1% decision-probability contour.

Figure 5 illustrates the statistical behavior of the MR-based decision probability as a function of the threshold $K_{MR}$ and the interference level. A similar trend can be observed, where the decision probability is relatively high for small $K_{MR}$ values and increases rapidly with the interference intensity. In homogeneous environments, selecting $K_{MR}=2.5$ ensures that the MR decision probability remains below 1%, effectively suppressing unnecessary variable-decision activation. Compared with the VI-based decision, the MR-based probability surface exhibits a steeper transition with respect to the interference level, indicating a higher sensitivity to interference clutter.

By jointly considering the false decision suppression capability in interference-free scenarios and the sensitivity of variable decision-making in the presence of interference, the threshold configuration of $K_{VI}=3$ and $K_{MR}=2.5$ is adopted for the DET-NTVI-CFAR detector in the subsequent experiments.

### 4.3. Effects of Parameters k, D, and N on Different Doppler-Spread Spectra

The performance of the DET-NTVI-CFAR detector is primarily determined by three parameters, namely the nonlinear exponent k, the accumulation window length D, and the number of reference cells N. Therefore, Monte Carlo simulations are employed to systematically investigate the effects of these parameters under different Doppler-spread conditions.

**Figure 6:**
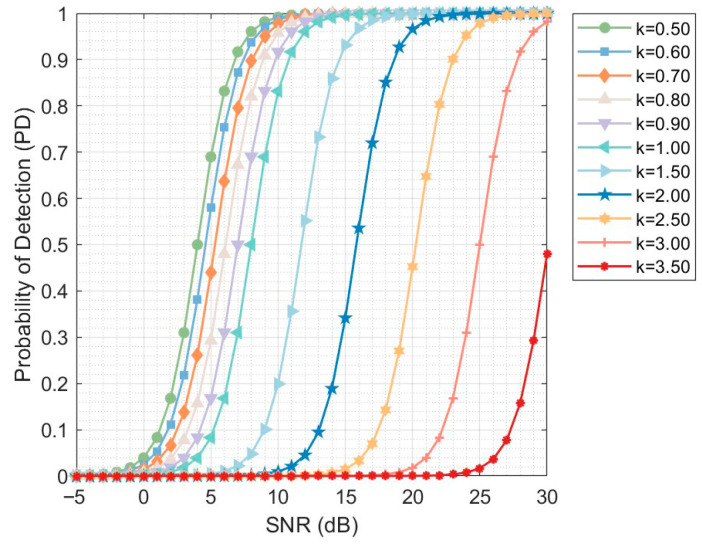
Detection probability versus SNR for different k.

The nonlinear exponent k determines the degree of nonlinear enhancement applied to the accumulated power statistic, and its primary effect lies in amplifying target features in the low SNR region. As illustrated in Figure 6, decreasing k causes the probability of detection (PD) curves to shift toward lower SNR region, indicating a significant improvement in the detector’s capability to identify weak targets. When k is relatively large, the nonlinear transformation leads to excessive compression of high-amplitude samples. Thus, the amplitude separability between the target echo and the background clutter is significantly degraded. As a consequence, the detection probability remains close to zero over a wide SNR range. As k decreases, the detection performance at low SNRs improves markedly. However, the performance gain gradually diminishes as k continues to decrease. Once k falls below a certain threshold, further reductions provide only marginal improvement. Specifically, the leftward shift of the PD curves begins to saturate, and in high-SNR conditions, the detection probabilities for different values of k converge to unity.

Further inspection of Figure 6 indicates that the enhancement effect becomes limited when 0.5≤k≤0.7, suggesting that the detection performance has entered a saturation region. Considering the trade-off among the performance enhancement in low-SNR region, numerical stability and implementation complexity, a fixed value of k=0.6 is adopted in this work.

**Figure 7:**
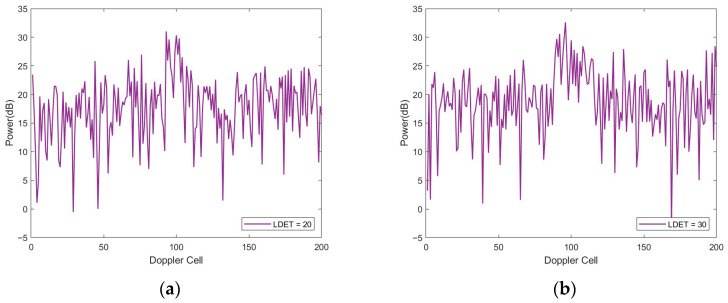
Representative simulated Doppler spectral profiles under different Doppler-spread conditions. (**a**) Simulated Doppler spectrum with $L_{DET}$ = 20; (**b**) Simulated Doppler spectrum with $L_{DET}$ = 30.

Figure 7a,b present representative Doppler spectral profiles generated for two cases with $L_{DET}=20$ and $L_{DET}=30$, respectively.

**Figure 8:**
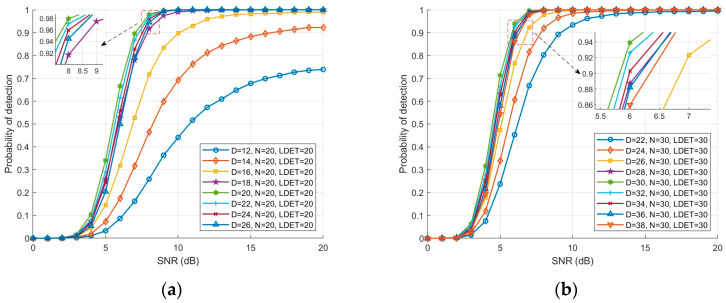
Detection probability versus SNR of the DET-NTVI-CFAR detector under different accumulation lengths D. (**a**) Detection probability comparison with N = 20 and $L_{DET}=20$; (**b**) Detection probability comparison with N = 30 and $L_{DET}=30$.

With the number of reference cells N kept fixed, Figure 8a,b illustrate the detection probability of the DET-NTVI-CFAR detector as a function of SNR for different accumulation lengths D. The results indicate that when the accumulation length D is smaller than the target Doppler extent $L_{DET}$, the detection probability increases with SNR but remains relatively low, suggesting that the target energy is not fully accumulated and the detection capability is limited. When D is equal to $L_{DET}$, the detection probability curve reaches its optimum, demonstrating that the accumulation length matches the Doppler extent characteristics of the target, thereby maximizing energy accumulation and detection performance. Furthermore, it can be observed that when D is larger than $L_{DET}$, the reduction in detection performance is limited. In particular, at high SNR, the curve is nearly saturated, indicating that the detector exhibits a certain robustness to overestimation of the accumulation length. The inset further shows that, in the low SNR region, variations in accumulation length still affect the detection probability. However, the overall trend remains stable, which validates the adaptability and reliability of the proposed algorithm in selecting the accumulation length.

**Figure 9:**
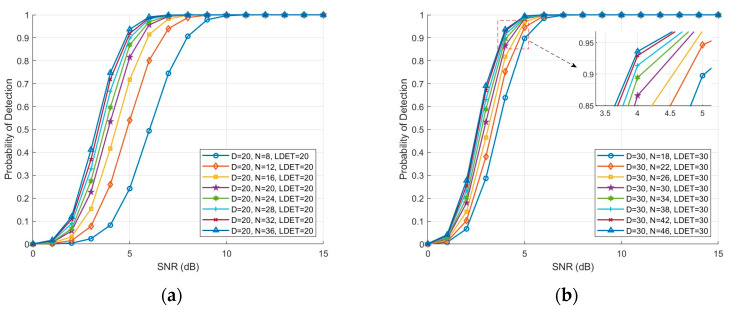
Detection probability versus SNR of the DET-NTVI-CFAR detector under different numbers of reference cells N. (**a**) Detection probability comparison with D = 20 and $L_{DET}=20$; (**b**) Detection probability comparison with D = 30 and $L_{DET}=30$.

With the optimal setting ${D=L}_{DET}$ fixed, Figure 9a,b illustrate the effect of different numbers of reference cells N on the detection probability as a function of SNR. As shown in Figure 9a, when ${D=L}_{DET}=20$, increasing N from 8 to 36 results in a leftward shift of the detection probability curves, indicating a significant improvement in detection performance at the same SNR. For instance, at SNR=6 dB, the detection probability is approximately 0.4 for N=8, while it approaches 1 for N=36. Figure 9b corresponds to ${D=L}_{DET}=30$, where increasing N from 18 to 46 similarly enhances the detection probability monotonically with SNR. These results indicate that the number of reference cells N has a significant impact on the statistical stability of the threshold estimation. A small N leads to large fluctuations in the detection threshold, resulting in unstable false alarm probabilities. In contrast, a large N introduces noise samples far from the CUT, which raises the detection threshold, reduces sensitivity to weak targets, and increases computational complexity. Therefore, a moderate N should be chosen to achieve a balance between statistical stability, high detection probability and reliable false alarm control.

The simulation results demonstrate that the proposed DET-NTVI-CFAR detector achieves a favorable trade-off between detection performance, algorithmic stability and robustness. Overall, the parameter k primarily influences the location and steepness of the transition region of the detection probability from low to high values, while its impact on the asymptotic detection performance in the high-SNR region is limited. When selecting the accumulation window length D, its value should be comparable to or slightly larger than the actual Doppler spread of the target to ensure satisfactory detection performance and to avoid degradation caused by parameter mismatch. Under ideal interference-free conditions, increasing the number of reference cells N can theoretically enhance detection capability. However, in practical engineering applications, parameter configuration requires careful consideration of computational complexity and system resource constraints, so as to achieve a balanced trade-off among detection performance, stability, and resource efficiency.

### 4.4. Detection Performance in Different Clutter Environments

In this section, the performance of the proposed DET-NTVI-CFAR detector is evaluated under three representative scenarios and compared with several existing CFAR methods. The comparison methods include the recently reported enhanced detector [33], the ABC detector [34], and conventional point-target CFAR algorithms, such as CA-CFAR, GO-CFAR, SO-CFAR, OS-CFAR and VI-CFAR.

To ensure a fair comparison, the accumulation window length D and the number of reference cells N are uniformly configured as 12 and 16, respectively. For the enhanced detector, the parameters are configured as D=12, m=24, and $2n_{1}=32$. The ABC detector adopts $N_{v}=1$, $L_{1}=3$, $L_{2}=4$, and k=4. All conventional point-target CFAR methods employ 32 reference cells. Using Monte Carlo simulations with a large number of random realizations, the detection probability and false alarm probability of each method are systematically evaluated under different SNR conditions. The simulation results and corresponding analyses are presented as follows.

#### 4.4.1. Homogeneous Environment

**Figure 10:**
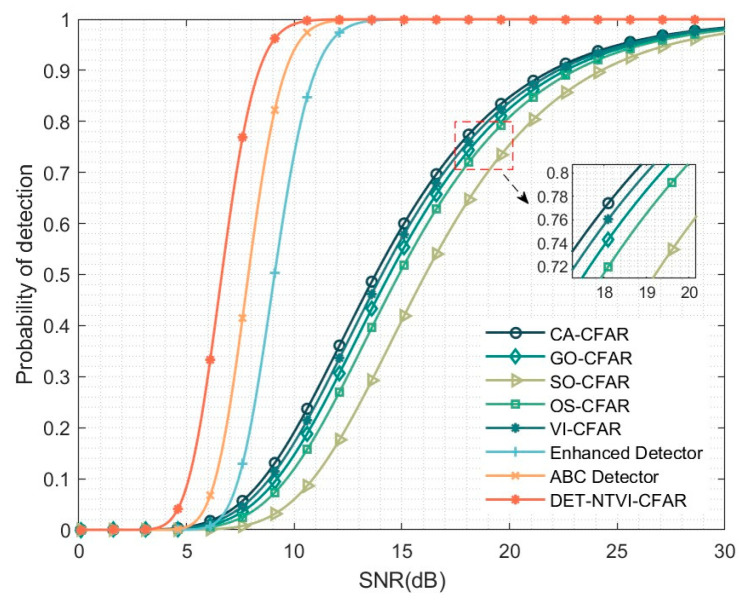
Detection performance of the DET-NTVI-CFAR compared with other CFAR methods under homogeneous environment.

Figure 10 shows the detection performance comparison of different CFAR detectors under homogeneous background conditions. The DET-NTVI-CFAR method consistently outperforms others in detection accuracy across the entire SNR range, with significant improvements in low and medium SNR regions. These results indicate that, by appropriately selecting the parameters, DET-NTVI-CFAR is able to effectively regulate the detection threshold, thereby significantly enhancing weak-target detectability while maintaining strict false alarm control.

In addition, although the detection probability curves of DET-NTVI-CFAR and the ABC detector exhibit similar overall trends, DET-NTVI-CFAR generally achieves a higher detection probability under the same SNR conditions. This difference in performance primarily stems from the unique structures of the reference cells and accumulation units used by the two methods. In comparison, under the experimental conditions analyzed in this study, the enhanced detector displays slightly lower detection performance when compared to DET-NTVI-CFAR. From a structural perspective, the performance improvement in homogeneous environments indicates that Doppler-domain energy integration combined with nonlinear tail enhancement increases the statistical separability between target returns and background noise while preserving CFAR stability. Overall, DET-NTVI-CFAR effectively leverages the statistical characteristics of VI-CFAR and demonstrates superior and consistent detection capabilities in homogeneous background environments.

#### 4.4.2. Interference Environment

**Figure 11:**
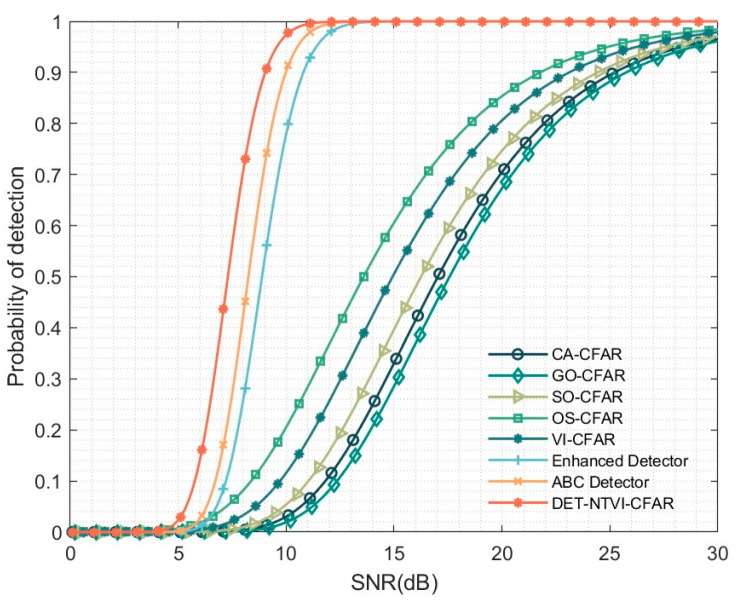
Detection performance of the DET-NTVI-CFAR compared with other CFAR methods in an interference environment.

To further evaluate the robustness of different detectors under interference conditions, this study constructs an interfering target scenario. Figure 11 compares the detection performance of various CFAR detectors under this interference scenario. The proposed DET-NTVI-CFAR detector consistently demonstrates superior performance over competing approaches throughout the full SNR range. This superior performance is mainly attributed to the introduction of a nonlinear transformation and a VI decision mechanism, which effectively suppresses the influence of abnormally large reference samples induced by the interfering target on background estimation. Consequently, the detection threshold remains stable, which results in a substantial improvement in detection probability.

Further comparison indicates that, although the ABC detector exhibits a certain degree of robustness in interference environments, its detection performance remains inferior to that of DET-NTVI-CFAR under the same SNR conditions. This performance gap primarily stems from the stronger adaptability of DET-NTVI-CFAR in reference cell statistical modeling and decision-making, which enables more effective mitigation of the impact of strong interference components on the detection threshold. The enhanced detector demonstrates a certain level of stability under interference conditions, with its detection performance generally lying between that of conventional CFAR methods and DET-NTVI-CFAR. However, its ability to suppress strong interfering targets remains limited. These results confirm that the VI-driven adaptive branch selection effectively prevents threshold overestimation caused by contaminated reference cells, thereby preserving detection sensitivity under multitarget interference conditions. Overall, simulation results indicate that the proposed DET-NTVI-CFAR detector maintains stable and reliable detection performance.

#### 4.4.3. Edge Clutter Environment

To comprehensively evaluate the performance of the detectors under clutter transition conditions, two distinct edge clutter scenarios are considered, namely the abrupt edge scenario and the gradual edge scenario. In both scenarios, the clutter background follows the same statistical distribution as in the homogeneous case, while the average clutter power varies along the sliding window direction.

In the abrupt edge scenario, the clutter region is divided into two segments with distinct power levels of 16 dB and 24 dB. The clutter power exhibits a step change at a fixed boundary. As the sliding reference window crosses this boundary, high-power clutter cells suddenly enter the reference window, resulting in a rapid change in the estimated background level and potentially generating a peak in the false alarm probability. In the gradual edge scenario, the clutter power increases linearly from 16 dB to 24 dB within a transition region. As the reference window moves across this region, the proportion of high-power clutter cells within the window increases progressively, leading to a continuously varying background estimate.

**Figure 12:**
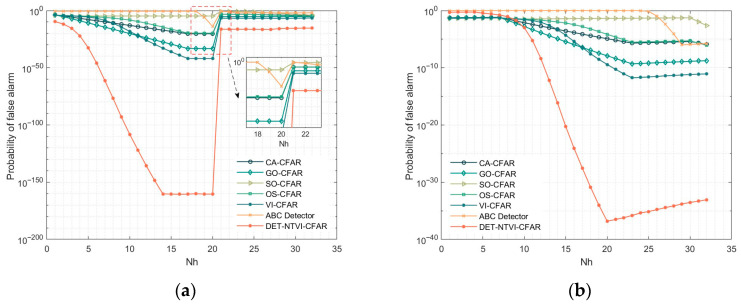
Detection performance of the DET-NTVI-CFAR compared with other CFAR methods in edge clutter environment. (**a**) Abrupt edge clutter environment; (**b**) Gradual edge clutter environment.

In Figure 12, the horizontal axis $N_{h}$ denotes the number of high-power clutter cells contained in the reference window, which reflects the degree of contamination of the reference window by high-power clutter samples. The simulation results, shown in Figure 12a, indicate that the proposed DET-NTVI-CFAR detector achieves the lowest false alarm probability peak under abrupt edge conditions and effectively suppresses the false alarm risk caused by sudden power variations within the reference window. In contrast, the SO-CFAR and ABC methods exhibit noticeably higher false alarm probabilities. In the gradual edge scenario, the simulation results in Figure 12b demonstrate that the DET-NTVI-CFAR detector maintains a relatively low and stable false alarm probability throughout the entire gradual edge region, whereas the other methods exhibit consistently higher false alarm probabilities. Combining the results from both abrupt and gradual edge scenarios indicates that the proposed DET-NTVI-CFAR detector effectively suppresses false alarm peaks and maintains stable performance under varying clutter conditions. These results indicate that the statistically controlled threshold formulation, together with adaptive sub-algorithm switching, enhances robustness against background power transitions while preserving consistent false alarm regulation.

### 4.5. Discussion on AI-Based Detection Methods

Recent AI-based (learning-based) range–Doppler processing detectors can be broadly categorized into two types: (1) CNN-based approaches that treat the RDM as an image and perform target detection via classification, regression, or pixel-level prediction; (2) Transformer-based approaches that exploit global context modeling to improve robustness in complex backgrounds. By learning discriminative representations directly from data, these methods often achieve strong performance, particularly under low-SNR conditions.

However, learning-based detectors typically depend on: (1) sufficiently large and reliably annotated datasets; (2) reproducible training, validation, and test data splits; (3) consistent preprocessing pipelines and hyperparameter tuning strategies. Their performance is highly sensitive to the labeled data and training protocols, and additional operating-point calibration is often required to maintain a stable false alarm level under domain shifts or scene changes. Therefore, a rigorous and fair comparison between analytically designed CFAR detectors and learning-based methods is non-trivial. For DETs, dense labeling is particularly challenging because the target return spans multiple Doppler bins and may partially overlap with clutter and other targets, which can introduce additional subjectivity and dataset-dependent bias. Without a standardized dataset and protocol, the performance of learning-based methods may be dominated by data preparation choices rather than the intrinsic capability of the detectors, thus leading to potentially misleading conclusions.

Importantly, the primary goal of this paper is to develop a CFAR framework with analytically tractable false alarm control (i.e., an explicit relationship between the preset false alarm probability and the threshold factor). This property provides interpretability and stable CFAR detection under varying background conditions, while many learning-based detectors do not inherently guarantee CFAR characteristics and often require additional calibration to maintain a consistent operating point under domain shifts. Future work will establish a unified benchmark using the same DET datasets (or publicly available datasets when feasible) and will incorporate representative learning-based detectors as additional baselines. We will also investigate hybrid designs that combine learning-based detection methods with the analytically derived CFAR thresholding presented in this paper, aiming to leverage data-driven representation learning while preserving explicit and reliable false alarm control.

## 5. Field Test Results

In this section, the detection performance of the proposed DET-NTVI-CFAR detector is evaluated through field experiments.

### 5.1. Experimental Platform and Signal Processing Chain

The experimental platform consists of an AWR1642 mm-wave radar (Texas Instruments, Dallas, TX, USA) and a DCA1000 data capture board (Texas Instruments, Dallas, TX, USA) for raw data acquisition. The radar operates in FMCW mode and transmits LFM chirps within each frame. The reflected echoes are received by the radar antenna and mixed with the LO signal to generate IF signals after low-pass filtering. The digitized IF data are recorded through the DCA1000 capture board and processed offline. Static clutter suppression is first applied to mitigate strong zero-Doppler components caused by stationary objects. Range processing is then performed via fast-time FFT to obtain the range spectrum for each chirp. Subsequently, slow-time FFT across multiple chirps is carried out to generate the RDM, where each cell corresponds to a specific range and Doppler frequency bin. All signal processing and algorithm implementation were performed in MATLAB R2022b (MathWorks, Natick, MA, USA).

The measured RDMs exhibit Doppler-extended characteristics of targets, in which target energy occupies multiple adjacent Doppler bins due to non-rigid motion and micro-Doppler effects. The proposed sliding accumulation and nonlinear transformation are directly applied to the generated RDM to construct the test statistic and determine the adaptive detection threshold.

### 5.2. Field Test Scenario and Detection Performance

**Figure 13:**
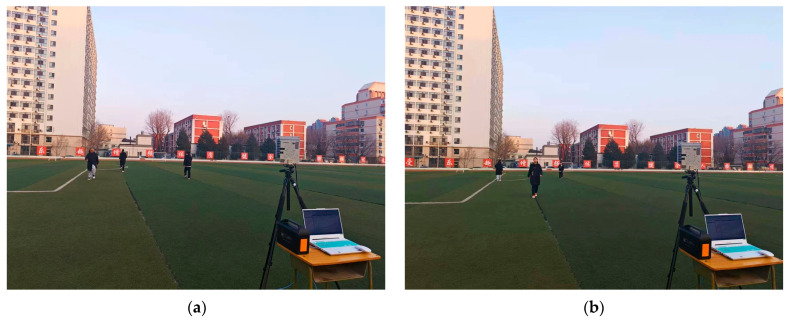
Field test scenarios. (**a**) Two pedestrians walk at the same range with opposite radial velocities, and a third pedestrian walks at a farther range; (**b**) Three pedestrians walk at different ranges with mixed radial velocities.

Field experiments were conducted in an open outdoor environment to evaluate the detection performance under multitarget conditions. The field test scenarios are presented in Figure 13. The radar system is mounted on a tripod and connected to a data acquisition and processing terminal for continuous collection of echo signals. Within an effective detection range of approximately 20 m in front of the radar, three pedestrians move at approximately ±1 m/s. Among the pedestrians, some are moving toward the radar, corresponding to negative radial velocities, while others are moving away from the radar, resulting in positive radial velocities. The acquired radar signals are subjected to standard preprocessing. Subsequently, a slow-time FFT along multiple chirps is applied to obtain 128 Doppler bins, thereby forming the RDM.

**Figure 14:**
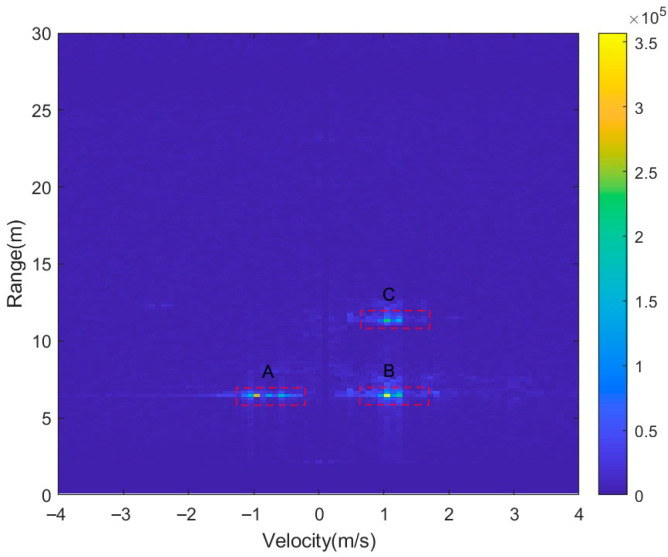
RDM when two pedestrians walk at the same range with opposite radial velocities, and a third pedestrian walks at a farther range.

Figure 14 illustrates the processed RDM corresponding to the three-target field test scenario shown in Figure 13a. At approximately 7 m, two pedestrian targets are observed, with one located in the positive Doppler region and the other in the negative Doppler region. These correspond to opposite radial velocity directions and are labeled as B and A, respectively. Additionally, a third pedestrian target (labeled C) is observed at approximately 12 m. Its energy is mainly concentrated in the positive Doppler region, indicating a positive radial velocity. Due to the non-rigid motion associated with human walking and the effects of micro-Doppler, all three targets exhibit varying degrees of spectral broadening in the Doppler dimension. This results in partial energy overlap within the local range-Doppler cells, which poses challenges for the stability and reliability of subsequent detection decisions. Based on the measured RDM and the Doppler resolution, the Doppler extent of the targets is estimated from the width of the dominant cluster, yielding $L_{DET}=12$. To mitigate the influence of additional Doppler-extended cells while preserving a high detection probability, the parameters of the DET-NTVI-CFAR detector are set to D=12 and N=16.

**Figure 15:**
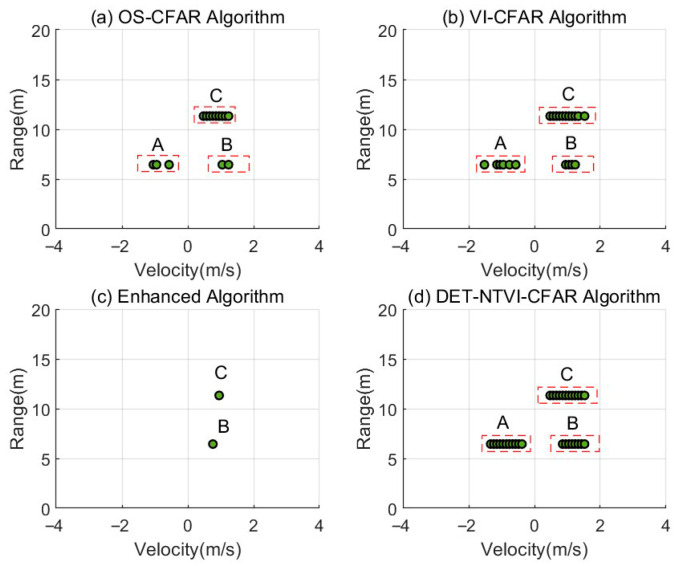
CFAR detection results when two pedestrians walk at the same range with opposite radial velocities, and a third pedestrian walks at a farther range. Targets A and B denote the two pedestrians at the same range with opposite radial velocities, while target C denotes the pedestrian at the farther range.

Figure 15 presents a comparison of the detection results obtained by different CFAR methods in this multitarget scenario, including the conventional OS-CFAR, VI-CFAR, the enhanced detector, and the proposed DET-NTVI-CFAR method. The detection results indicate that both OS-CFAR and VI-CFAR can identify the three pedestrian targets to some degree. However, their detection points are relatively scattered in the spatial domain. This is especially evident when targets at the same range but with different velocities are present. In such cases, local energy imbalances can influence the detection results, making it difficult to achieve continuous and stable responses for the targets. In contrast, the enhanced detector primarily detects the energy-dominant component, reliably identifying only the strongest targets. As a result, it lacks sufficient sensitivity to weaker targets at the same range, leading to missed detections. The proposed DET-NTVI-CFAR method demonstrates superior detection stability and consistency in this three-target scenario. It is capable of simultaneously detecting targets A and B at the same range with opposite radial velocities, while also providing stable and continuous responses for target C located at a different range. Moreover, DET-NTVI-CFAR generates denser and more spatially continuous detection points within the target regions, enabling a more complete characterization of the energy distribution of extended targets in the range-Doppler plane.

**Figure 16:**
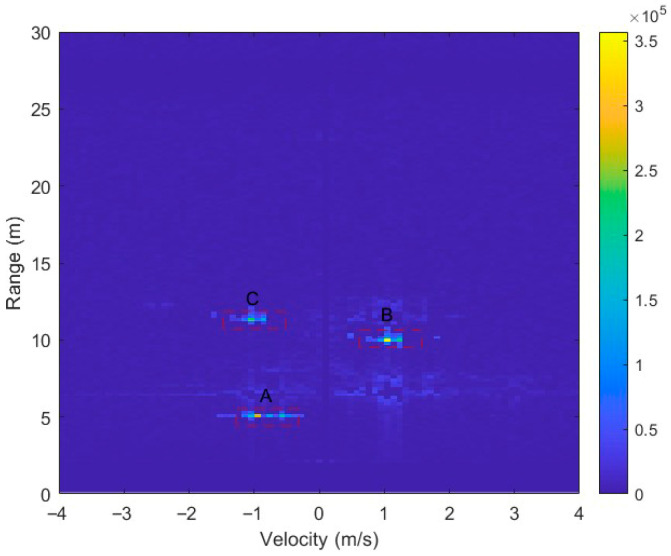
RDM when three pedestrians walk at different ranges with mixed radial velocities.

To validate the robustness of the proposed method under different geometric distributions, another three-target configuration shown in Figure 13b is examined. In this configuration, three targets are located at approximately 5 m (target A), 10 m (target B), and 12 m (target C). Target B is mainly distributed in the positive Doppler region, while targets A and C are concentrated in the negative Doppler region. The corresponding RDM is presented in Figure 16. Compared with the same-range scenario in Figure 14, the targets are now separated in range. Each target still exhibits noticeable Doppler-domain spectral broadening due to human micro-motion.

**Figure 17:**
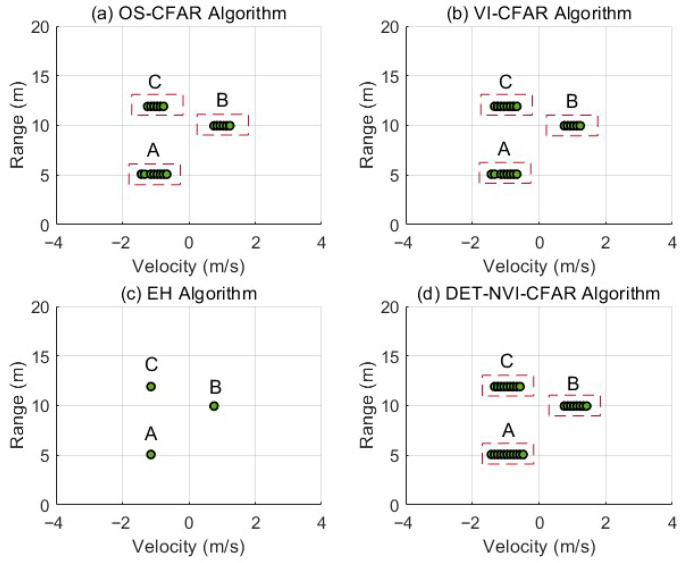
CFAR detection results when three pedestrians walk at different ranges with mixed radial velocities. Targets A, B, and C correspond to the pedestrians located at approximately 5 m, 10 m, and 12 m, respectively.

As shown in Figure 17, OS-CFAR and VI-CFAR can detect the three targets. However, the detection points remain relatively sparse and less structured within the target regions. The enhanced detector mainly responds to local energy peaks and yields limited spatial coverage. In contrast, the proposed DET-NTVI-CFAR method generates more continuous and spatially coherent detection point sets. This experiment further confirms that the proposed DET-NTVI-CFAR framework maintains stable detection performance under different spatial configurations, including both same-range and different-range multitarget scenarios.

Further analysis from the perspective of the spatial distribution characteristics of the detection points indicates that the continuous detection point sets produced by DET-NTVI-CFAR provide more structured observational information for subsequent high-level processing. During the angle-of-arrival estimation and point cloud clustering stages, detection points with higher spatial consistency contribute to improved angle estimation accuracy and clustering stability, while effectively lowering the risks of false associations and missed detections. During the target tracking stage, continuous and stable detection responses form coherent observation trajectories. Such trajectories enhance the robustness of the tracking system under noise fluctuations and short-term occlusions, thereby improving the continuity and reliability of the tracking results.

## 6. Conclusions

This paper proposes and investigates a DET-NTVI-CFAR detector. By exploiting the echo characteristics of DET, the proposed method accumulates target energy along the Doppler dimension and employs a nonlinear transformation to enhance the tail behavior of the statistic, leading to improved detection performance. To facilitate the adaptive threshold construction of the DET-NTVI-CFAR detector, three sub-detection methods, namely DET-TCA-CFAR, DET-TSO-CFAR, and DET-TGO-CFAR, are formulated. The corresponding threshold factors and expressions for the false alarm probability are analytically derived. These expressions provide a theoretical basis for threshold design and parameter configuration. In addition, Monte Carlo simulations are conducted to systematically analyze the relationship between detection performance and key parameters, including the nonlinear exponent, accumulation length, and the number of reference cells. Performance evaluations conducted in homogeneous, interference-target, and clutter-edge environments indicate that the proposed DET-NTVI-CFAR detector maintains stable, reliable detection performance under complex background conditions. Furthermore, field experiments confirm that the proposed DET-NTVI-CFAR method is applicable to practical FMCW radar scenarios, yielding more continuous and denser target detections while preserving stable performance. As the Doppler extent or environmental complexity increases, the configuration of parameters and the decision-making process of the detector become more complex. Therefore, finding ways to reduce implementation complexity while maintaining detection performance remains a significant area for future research.

## Figures and Tables

**Table 1:** DET-NTVI-CFAR adaptive threshold Selection Strategy.

Non-Homogeneity of Reference Matrix A	Non-Homogeneity of Reference Matrix B	Difference in Mean Values	DET-NTVI-CFAR Threshold	CFAR Strategy
NO	NO	NO	$\alpha_{2N}^{TCA}\cdot sum_{AB}$	DET-TCA
NO	NO	YES	$\alpha_{N}^{TGO}\cdot \max(sum_{A},sum_{B})$	DET-TGO
YES	NO	—	$\alpha_{N}^{TCA}\cdot sum_{B}$	DET-TCA
NO	YES	—	$\alpha_{N}^{TCA}\cdot sum_{A}$	DET-TCA
YES	YES	—	$\alpha_{N}^{TSO}\cdot \min(sum_{A},sum_{B})$	DET-TSO

## Data Availability

The original contributions presented in this study are included in the article. Further inquiries can be directed to the corresponding author.

## References

[1] Li F., Rao P., Sun W., Su Y., Chen X. (2025). A New Motion Feature-Enhanced Multiframe Spatial–Temporal Infrared Target Detection Network. IEEE Trans. Geosci. Remote Sens..

[2] Guo Z.-X., Bai X.-H., Shui P.-L., Wang L., Su J. (2023). Fast Dual Trifeature-Based Detection of Small Targets in Sea Clutter by Using Median Normalized Doppler Amplitude Spectra. IEEE J. Sel. Top. Appl. Earth Obs. Remote Sens..

[3] Wang D., Zhang Q., Xu Y., Zhang J., Du B., Tao D. (2023). Advancing Plain Vision Transformer Toward Remote Sensing Foundation Model. IEEE Trans. Geosci. Remote Sens..

[4] Li Y., Gu C., Mao J. (2022). 4-D Gesture Sensing Using Reconfigurable Virtual Array Based on a 60-GHz FMCW MIMO Radar Sensor. IEEE Trans. Microw. Theory Tech..

[5] Haider A., Eryildirim A., Pigniczki M., Haas L., Schlager B., Zeh T. (2025). Modeling and Simulation of Automotive FMCW RADAR Sensor for Environmental Perception. IEEE Open J. Intell. Transp. Syst..

[6] Li T., Peng D., Shi S. (2022). Outlier-Robust Superpixel-Level CFAR Detector With Truncated Clutter for Single Look Complex SAR Images. IEEE J. Sel. Top. Appl. Earth Obs. Remote Sens..

[7] Ehsanfar S., Bazzi A., Mößner K., Chafii M. Hypothesis Testing on FMCW and OFDM for Joint Communication and Radar in IEEE 802.11bd. Proceedings of the 2023 IEEE International Conference on Communications Workshops (ICC Workshops).

[8] Delamou M., Bazzi A., Chafii M., Amhoud E.M. Deep Learning-based Estimation for Multitarget Radar Detection. Proceedings of the 2023 IEEE 97th Vehicular Technology Conference (VTC2023-Spring).

[9] Carrera E.V., Lara F., Ortiz M., Tinoco A., León R. Target Detection using Radar Processors based on Machine Learning. Proceedings of the 2020 IEEE ANDESCON.

[10] Yavuz F., Kalfa M. Radar Target Detection via Deep Learning. Proceedings of the 2020 28th Signal Processing and Communications Applications Conference (SIU).

[11] Zhang Z., Lai H., Huang D., Fang X., Zhou M., Zhang Y. (2024). RETA: 4D Radar-Based End-to-End Joint Tracking and Activity Estimation for Low-Observable Pedestrian Safety in Cluttered Traffic Scenarios. IEEE Trans. Intell. Transp. Syst..

[12] Nekounam N., Khan Z., Koskinen L. (2025). Efficient SVD Techniques to Overcome Interference and Obstacle Challenges for Micro-Doppler Extraction in FMCW Radars. IEEE Sens. J..

[13] Fang X., Li J., Zhang Z., Xiao G. (2022). FMCW-MIMO Radar-Based Pedestrian Trajectory Tracking Under Low- Observable Environments. IEEE Sens. J..

[14] Tang M., Rong Y., De Maio A., Chen C., Zhou J. (2019). Adaptive Radar Detection in Gaussian Disturbance With Structured Covariance Matrix via Invariance Theory. IEEE Trans. Signal Process..

[15] Meziani H.A., Soltani F. (2006). Performance analysis of some CFAR detectors in homogeneous and non-homogeneous pearson-distributed clutter. Signal Process..

[16] Hansen V.G., Sawyers J.H. (1980). Detectability Loss Due to “Greatest Of” Selection in a Cell-Averaging CFAR. IEEE Trans. Aerosp. Electron. Syst..

[17] Weiss M. (1982). Analysis of Some Modified Cell-Averaging CFAR Processors in Multiple-Target Situations. IEEE Trans. Aerosp. Electron. Syst..

[18] Zaimbashi A., Taban M.R., Nayebi M.M. Order Statistic and Maximum Likelihood Distributed CFAR Detectors in Weibull Background. Proceedings of the 2007 IEEE Radar Conference.

[19] Barboy B., Lomes A., Perkalski E. (1986). Cell-averaging CFAR for multiple-target situations. IEE Proc. F-Commun. Radar Signal Process..

[20] Smith M.E., Varshney P.K. (2000). Intelligent CFAR processor based on data variability. IEEE Trans. Aerosp. Electron. Syst..

[21] Lu S., Sun X., Ding F., Li R. Robust Distributed Sonar CFAR Detection Based on Modified VI-CFAR Detector. Proceedings of the 2019 International Conference on Control, Automation and Information Sciences (ICCAIS).

[22] Cao Z., Li J., Song C., Xu Z., Wang X. (2021). Compressed Sensing-Based Multitarget CFAR Detection Algorithm for FMCW Radar. IEEE Trans. Geosci. Remote Sens..

[23] Cao Z., Li J., Song C., Xu Z., Wang X. A Novel CFAR Algorithm for Multi-target Detection with FMCW Radar. Proceedings of the GLOBECOM 2020—2020 IEEE Global Communications Conference.

[24] Orlando D., Ricci G. (2025). A New CFAR Detector Based on the EM Algorithm. IEEE Signal Process. Lett..

[25] He X., Xu Y., Liu M., Hao C. (2023). Inhomogeneity Suppression CFAR Detection Based on Statistical Modeling. IEEE Trans. Aerosp. Electron. Syst..

[26] Zhu X., Tu L., Zhou S., Zhang Z. (2022). Robust Variability Index CFAR Detector Based on Bayesian Interference Control. IEEE Trans. Geosci. Remote Sens..

[27] Wang X., Li Y., Zhang N. (2023). A Robust Variability Index CFAR Detector for Weibull Background. IEEE Trans. Aerosp. Electron. Syst..

[28] Coluccia A., Fascista A., Ricci G. (2020). A k-nearest neighbors approach to the design of radar detectors. Signal Process..

[29] Liu K., Li Y., Wang P., Peng X., Liao H., Li W. (2023). A CFAR Detection Algorithm Based on Clutter Knowledge for Cognitive Radar. IEICE Trans. Fundam. Electron. Commun. Comput. Sci..

[30] Zhao Z., Wang H., Cao L., Wang D., Fu C. (2024). Doppler-Spread Target Summation Variability Index CFAR Detector for FMCW Radar. IEEE Sens. J..

[31] Yang J., Yi J., Wan X., Cheng F. (2022). Rank Test-Based Scattering Center Number Estimation in Extended Radar-Target Detection. IEEE Commun. Lett..

[32] Ye Y., Deng Z., Pan P., Ma W., Huang X. (2022). Doppler-Spread Targets Detection for FMCW Radar Using Concurrent RDMs. IEEE Trans. Veh. Technol..

[33] Zhang W., Li H., Sun G., He Z. (2019). Enhanced Detection of Doppler-Spread Targets for FMCW Radar. IEEE Trans. Aerosp. Electron. Syst..

[34] Wei Z., Li B., Feng T., Tao Y., Zhao C. (2023). Area-Based CFAR Target Detection for Automotive Millimeter-Wave Radar. IEEE Trans. Veh. Technol..

[35] Raman Subramanyan N., Kalpathi R.R., Vengadarajan A. (2019). Robust variability index CFAR for non-homogeneous background. IET Radar Sonar Navig..

[36] Ai J., Mao Y., Luo Q., Xing M., Jiang K., Jia L. (2021). Robust CFAR Ship Detector Based on Bilateral-Trimmed-Statistics of Complex Ocean Scenes in SAR Imagery: A Closed-Form Solution. IEEE Trans. Aerosp. Electron. Syst..

